# Whole-Genome Uterine Artery Transcriptome Profiling and Alternative Splicing Analysis in Rat Pregnancy

**DOI:** 10.3390/ijms21062079

**Published:** 2020-03-18

**Authors:** Kathirvel Gopalakrishnan, Sathish Kumar

**Affiliations:** 1 Department of Comparative Biosciences, School of Veterinary Medicine, University of Wisconsin, Madison, WI 53706, USA; 2 Department of Obstetrics and Gynecology, School of Medicine and Public Health, University of Wisconsin, Madison, WI 53792, USA; 3 Endocrinology-Reproductive Physiology Program, University of Wisconsin, Madison, WI 53715, USA

**Keywords:** pregnancy, uterine arteries, RNA-seq, alternative splicing, transcriptome

## Abstract

During pregnancy, the uterine artery (UA) undergoes extensive remodeling to permit a 20–40 fold increase in blood flow with associated changes in the expression of a multitude of genes. This study used next-gen RNA sequencing technology to identify pathways and genes potentially involved in arterial adaptations in pregnant rat UA (gestation day 20) compared with non-pregnant rat UA (diestrus). A total of 2245 genes were differentially expressed, with 1257 up-regulated and 970 down-regulated in pregnant UA. Gene clustering analysis revealed a unique cluster of suppressed genes implicated in calcium signaling pathway and vascular smooth muscle contraction in pregnant UA. Transcription factor binding site motif scanning identified C2H2 ZF, AP-2 and CxxC as likely factors functional on the promoters of down-regulated genes involved in calcium signaling and vascular smooth muscle contraction. In addition, 1686 genes exhibited alternative splicing that were mainly implicated in microtubule organization and smooth muscle contraction. Cross-comparison analysis identified novel genes that were both differentially expressed and alternatively spliced; these were involved in leukocyte and B cell biology and lipid metabolism. In conclusion, this first comprehensive study provides a valuable resource for understanding the molecular mechanism underlying gestational uterine arterial adaptations during pregnancy.

## 1. Introduction

Pregnancy is one of the most complex reproductive activities of mammals, wherein extensive changes, especially in the uterine arteries (UA), occur to permit the supply of nutrients to the placenta and developing fetus. The diameter of the UA increases by two to three fold from a non-pregnant to late pregnant state in humans and animals [1,2,3], with a striking 20–40-fold increase in UA blood flow [4,5]. To accommodate this increase in uterine blood flow, the uterine vasculature undergoes arterial wall expansion and hyperplasia, a process termed outward hypertrophic remodeling or arteriogenesis [6]. In addition to the structural changes, uterine vascular reactivity is also altered during pregnancy, with the general pattern reduced tone and enhanced vasodilation/blunted vasoconstriction [7]. These differences between pregnancy and non-pregnancy are tightly regulated by the external environment, various endocrine factors, and the expression of a large number of genes [8,9,10].

Studies have indicated that the differential expression of hormone receptor genes (estrogen [*ERα*, *ERβ*, *GPER*] [11,12], relaxin [*RXFP1*] [13], angiotensin [*AT1R*, *AT2R*] [14] and adrenomedullin [*CRLR*, *RAMP*]) [15], calcium and potassium channels [16,17], growth factor genes (vascular endothelial growth factor [*VEGF*], placental growth factor, insulin-like growth factor 1 [*IGF-I*] and bone morphogenetic protein 6 [*BMP6*], vasodilator genes (nitric oxide (endothelial nitric oxide synthase), and hydrogen sulfide [cystathionine β-synthase]) [3,18] in uterine vasculature during pregnancy significantly impact vascular remodeling and vasodilation. Furthermore, several other signaling pathways have been implicated in arterial remodeling, including matrix metalloproteinases (MMP) activation [19], adrenergic influences [20], toll-like receptors [21], cytoskeleton [22], including vimentin [23], and membrane-associated tyrosine kinases such as PYK2 [24]. These studies were conducted in isolation, and surprisingly, no studies have comprehensively examined global transcriptome and the associated pathways involved in normal gestational uterine vascular adaptations.

Another missing link is whether post-transcriptional regulation also contributes to the functions of key genes that modulate pregnancy mediated uterine vascular adaptations. Although the pre-mRNAs are transcribed from a single gene locus, through the alternative splicing, like differential exon inclusion or skipping, the pre-mRNAs can be spliced into different ways to generate divergent isoforms [25,26]. These new isoforms often result in interaction profiles that are divergent from the ‘canonical’ isoform [27]. There is only one study that discovered changes in alternative splicing of guanylyl cyclase, a major vasodilatory mediator, in the pregnant ovine UA [28]. Thus, whether the aberrant splicing of a network of genes occurs in the UA and if they relate to the gestational vascular adaptations has not been explored. Furthermore, alterations in gene expression or posttranscriptional modification have been shown to disturb various critical functions that lead to pregnancy disorders, including fetal growth restriction, gestational diabetes, and preeclampsia [29,30,31,32,33]. However, no studies are available to provide insight into the general vascular transcriptome of the normal term UA. The goal of the current study was to provide the first comprehensive assessment of global transcriptome changes, alternative splicing, and the potential pathways involved in normal gestational uterine vascular adaptations.

## 2. Results

### 2.1. Statistics of the UA Transcriptome

**Figure 1:**
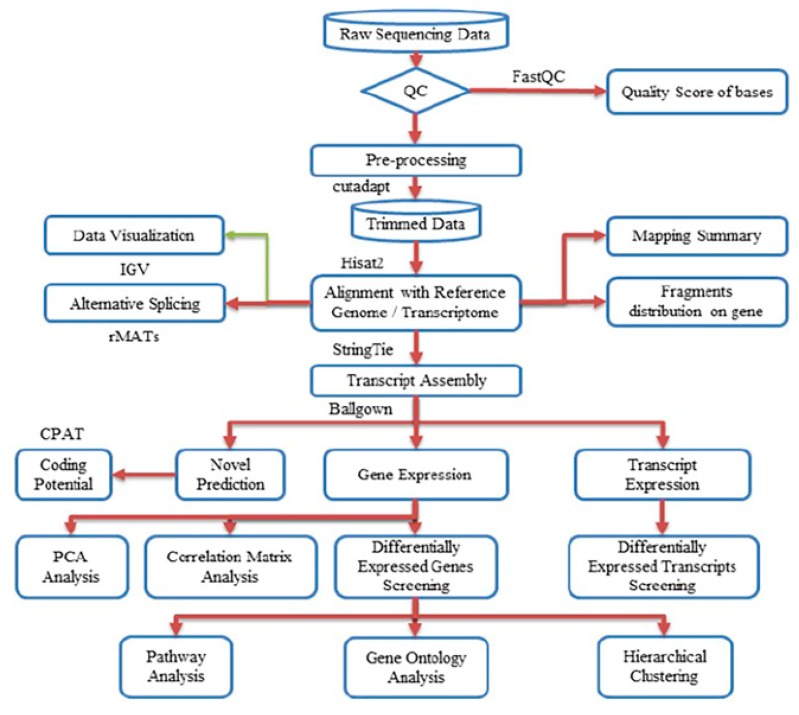
Workflow pipeline for sequencing, data processing, differential expression, alternative splicing, and bioinformatics analysis.

Next-gen RNA sequencing to elucidate the differential transcriptional regulation and alternative splicing between pregnant and non-pregnant UA yielded ~50–80 million 2  ×  125-bp paired-end reads with the Q30 quality score of 89%–90%. The quality scores for all samples are presented in Supplementary Table S1 and Figure S1. After preprocessing and trimming, ~90% of reads were uniquely mapped on the rat genome assembly Rnor_6.0 (GCA_000001895.4) using hisat2 (Table 1). The StringTie transcript assembly and ballgown expression analysis identified positive transcriptional signal (FPKM reads) for 14,308 genes and 17,950 transcripts in non-pregnant and 14,317 genes and 17,906 transcripts in pregnant UA (Supplementary Tables S2 and S3). The distinguishable gene expressions (for the genes that have the ANOVA *p*-value ≤ 0.05 on FPKM abundance) between groups were confirmed by the principal component analysis (PCA) analysis (Supplementary Figure S2A). Furthermore, the Pearson *R*^2^ score was over 0.92, showing a strong gene expression correlation between the biological samples in both groups (Supplementary Figure S2B). The complete workflow pipeline for sequencing, data processing, differential expression, alternative splicing, and bioinformatics analysis is presented in Figure 1.

### 2.2. Differentially-Expressed Genes (DEGs)

**Figure 2:**
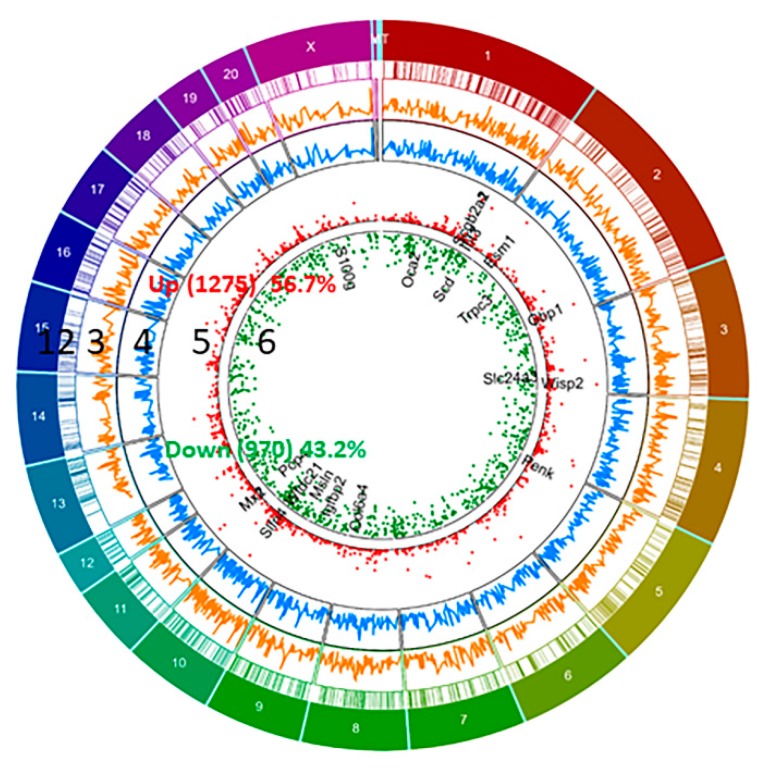
Circos plot of differentially expressed genes (DEGs) between pregnant and non-pregnant UA. DEGs more than 1.5 fold (*P* < 0.05, paired test) are presented as scatter plot expression levels are presented as green (Down, track 6) and red (Up, Track 5). FPKM of DEGs- non-pregnant (Blue, track 4) and pregnant (Orange, track 3) are presented as continuous lines. DEG locations are presented as rectangular lines (track 2) along with chromosomes (track 1).

We have identified differential expression of 2245 genes (with R package ballgown- fold change (cutoff 1.5), *p*-value (≤0.05) and FPKM (≥ 0.5 mean in one group)) between pregnant and non-pregnant UA; the majority of them (56.7%, 1275 genes) were upregulated and the remaining (43.2%, 970 genes) were downregulated in pregnant UA compared with non-pregnant UA (Figure 2, Supplementary Table S4).

### 2.3. qPCR Validation of Differentially Expressed Genes

**Figure 3:**
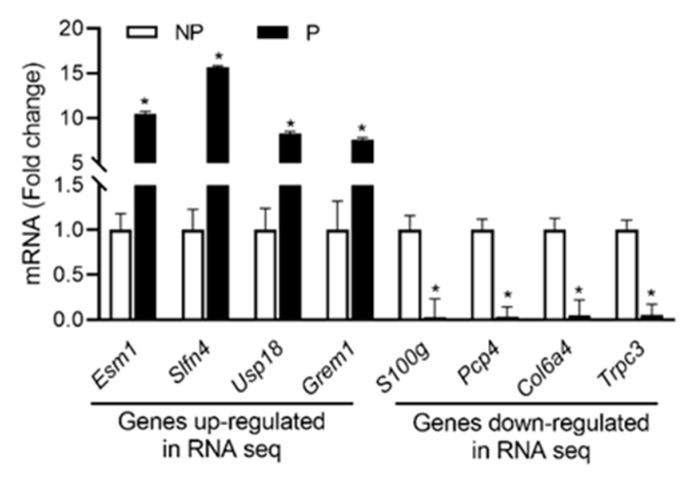
qRT-PCR validation of the four up and downregulated genes from RNA sequencing data. Quantitation of fold change was normalized relative to *Gapdh* levels. Values are given as means ± standard error of the mean (sem) of *n* = 3 per group (* *P* < 0.05). NP-non-pregnant; P-pregnant.

From RNA seq data sets, the expression profiles of the top four up- and down-regulated genes were validated using qPCR analysis. As shown in Figure 3, the expression of *Esm1*, *Slfn4*, *Usp18* and *Grem1* were upregulated, whereas the expression of *S100g*, *Pcp4*, *Col6a4* and *Trpc3* were downregulated in pregnant UA compared with non-pregnant UA consistent with the RNA-sequencing data.

### 2.4. Functional Enrichment, KEGG Pathway and Clustering Analysis of DEGs

**Figure 4:**
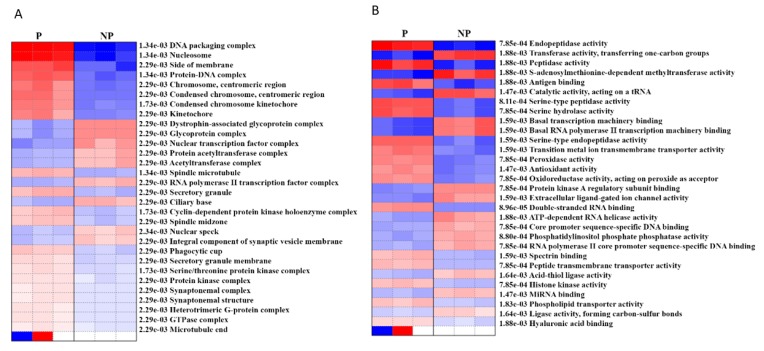
Pre-ranked gene set enrichment analysis (PGSEA) using gene ontology (GO) (**A**) cellular component and (**B**) molecular function. The corresponding activation (red)/suppression (blue) of components/functions are presented. The red and blue color gradients represent the level of activation/suppression, the higher level of activation is shown in dark red, and a higher level of suppression is shown in dark blue. NP-non-pregnant; P-pregnant.

In order to determine the cellular and molecular functions that are activated/ suppressed during pregnancy, we performed pre-ranked gene set enrichment analysis (PGESA) for Gene Ontology (GO). With regard to ‘cellular component,’ 21 processes were activated during pregnancy (the top three were DNA packaging complex, nucleosome, protein DNA complex with *p* = 1.34e−03, FDR = 0.05), while 9 were suppressed (top three were glycoprotein complex, nuclear transcription factor complex, acetyltransferase complex, *p* = 2.29e−03, FDR = 0.05). Likewise, with regard to ‘molecular function,’ 16 processes were activated [the top three (FDR = 0.05) were endopeptidase activity (*p* = 7.85e−04), peptidase activity (*p* = 1.88e−03), serine-type peptidase activity (*p* = 8.11e−04),] while 14 processes (FDR = 0.05) were suppressed (the top three were transferase activity (*p* = 1.88e−03), S-adenosylmethionine-dependent methyl-transferase activity (*p* = 1.88e−03) and catalytic activity, acting on a tRNA, (*p* = 1.47e−03)) (Figure 4, Supplementary Table S5).

**Figure 5:**
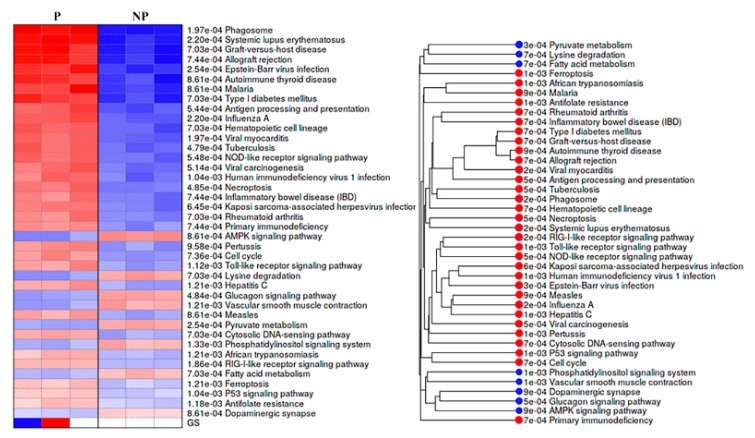
Pre- ranked gene set enrichment analysis (PGSEA) using KEGG pathways for the differentially expressed genes. Pathways activated are shown in red, and suppressed pathways shown in blue alongside the corresponding pathway tree are presented. The red and blue color gradients represent the level of activation/ suppression, the higher level of activation is shown in dark red and a higher level of suppression is shown in dark blue. NP-non-pregnant; P-pregnant.

KEGG pathways analysis showed that DEGs were engaged in 32 activated and 8 suppressed pathways in pregnant UA. The activated pathways mostly involved in immune system development and response with the top four (with lowest p values) being implicated in systemic lupus erythematosus (*p* = 2.20e−04), phagosome (*p* = 1.97e−040), graft-versus-host disease (*p* = 7.03e−04) and allograft rejection (*p* = 7.44e-04). In contrast, the suppressed pathways were widely distributed among various biological, cellular and molecular function with the top four (with lowest *p* values) being implicated in adenosine 5′monophosphate-activated protein kinase (AMPK) signaling pathway (*p* = 8.61e-04), lysine degradation (*p* = 7.03e-04), glucagon signaling pathway (*p* = 4.84e-04), and vascular smooth muscle contraction (*p* = 1.21e-03) (Figure 5, Supplementary Table S6).

**Figure 6:**
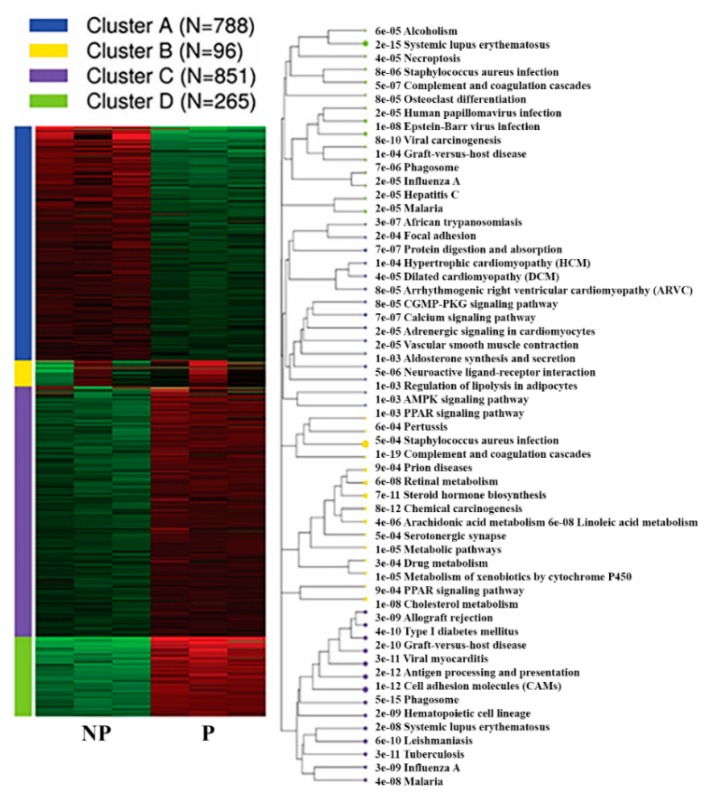
K-means Functional enrichment of differentially expressed genes in the pregnant UA by KEGG pathway alongside the corresponding pathway tree is presented. NP-non-pregnant; P-pregnant.

Furthermore, k-means clustering by integrated differential expression and pathway analysis (iDEP) identified four unique gene clusters. Notably, the genes within cluster C (851 genes) and D (265 genes) were upregulated, and include genes related to phagosome (35 genes), cell adhesion molecules (31 genes) and antigen processing and presentation (922 genes), systemic lupus erythematosus (18 genes), viral carcinogenesis (18 genes), and Epstein–Barr virus infection pathways (17 genes) (Figure 6, Table 2, Supplementary Table S7).

Vast majority of genes within cluster A (788 genes) were downregulated in UA during pregnancy. Among these, 25 genes were members of calcium signalling pathway (adj.*P* = 7.36E-07, *Cacna1h, Slc8a2, Ryr3, Adrb2, Agtr1a, Cacna1c, Tacr3, Ptger3, Grpr, Tacr2, Oxtr, Atp2a3, Adrb3, Mylk, Phkg1, Adra1a, Adra1d, Tnnc2, Pde1b, Adcy1, Cacna1e, Pln, Ryr2, Agtr1b and Chrm2*) and 18 genes were members of vascular smooth muscle contraction (adj.*P* = 1.95E-05, *Nppc, Ppp1r12a, Agt, Agtr1a, Cacna1c, Myh11, Adm, Actg2, Mylk, Pla2g5, Adra1a, Adra1d, Pla2g2a, Ppp1r12b, Adcy1, Mrvi1, Acta2, and Agtr1b*). Besides, genes within cluster A were also implicated in various pathways including neuroactive ligand-receptor interaction (33 genes), cGMP-PKG signaling pathway (19 genes), AMPK signaling pathway (14 genes) and ECM-receptor interaction (11 genes) (Figure 6, Supplementary Table S7).

### 2.5. Transcription Factor (TF) Motif Enrichment Analysis

Since we observed the distinguishable transcriptional signatures in pregnant UA, the identification of pregnancy-specific transcriptional regulators/ suppressers might be of relevance in gestational vascular adaptations. Thus, we conducted transcription factor (TF) binding motifs enrichment analysis on the (−300 bp from transcriptional start +1) promoter of DEGs within each cluster to evaluate which TFs are preferably binding in the promoter of upregulated or downregulated genes. Genes within each cluster showed enriched motif for specific TFs. Genes within cluster A showed enriched binding motifs for C2H2 ZF, AP-2 and CxxC. Similarly, genes within cluster C showed enriched binding motifs for ETS, REL and C2H2 ZF, and cluster D for IRF, STAT, CXC and Homeodomain members. Specifically, among the 25 downregulated genes in the calcium signalling pathway within cluster A, 6 genes (*Adra1a, Adrb3, Agtr1, Atp2a3, Oxtr* and *Grpr*) showed enriched binding motif for *Sp1* and *Crebp1* (*P* = 0.0002, FDR = 0.0005). Further, among the 18 genes downregulated in vascular smooth muscle contraction, 5 genes (*Adm, Adra1a, Agt, Agtr1* and *Pla2g2a*) showed enriched binding motif for *Tfap2a, sp1, Rela* and *Nfkb1* (*P* = 0.001, FDR = 0.007) (supplementary Table S8).

### 2.6. Alternative Splicing Patterns

Using splice junction counts as input, alternative splicing events (ASE) were investigated by rMATs paired model (v3.2.1 beta) to determine the extent of alternative splicing changes in the pregnant UA. Five basic and generally recognized alternative splicing modes were identified, including skipped exon (SE), mutually exclusion exons (MXE), alternative 5′ splice site (A5SS), alternative 3′ splice site (A3SS), and retained intron (RI). Rat UA exhibited 22,868 ASEs pertaining to 9971 genes with SE being the most common (86.1%; 19,707 SE events in 7747 genes) and A5SS being the least common (0.69%, 160 A5SS events in 152 genes (Table 3, Supplementary Table S9). Only two genes (*Zdhhc1* and *CcZ1b*) showed evidence of all five ASE types.

**Figure 7:**
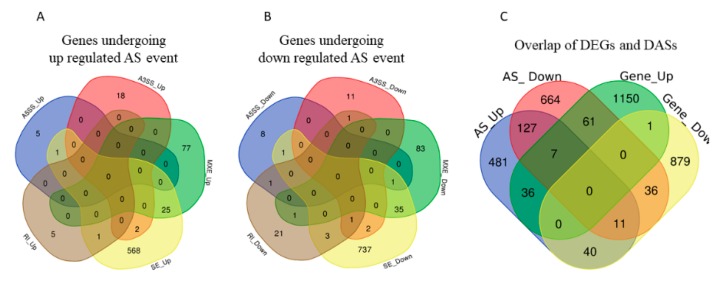
(**A**) Total number of genes with alternative splicing increase and (**B**) alternative splicing decrease and their types overlaps in pregnant compared with non-pregnant UA. A5SS: alternative 5′ splice, A3SS: alternative 5′ splice, MXE: Mutually exclusive exons, RI: retained intron, SE: Exon skipping. (**C**) The total number of both differentially expressed and alternatively spliced genes in pregnant UA.

**Figure 8:**
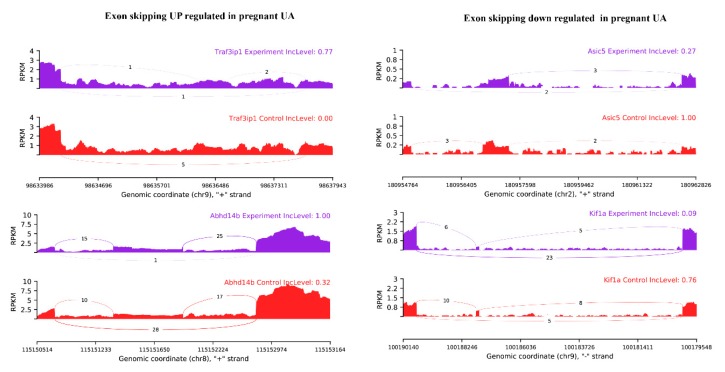
rMATS sashimi plot showing the up/down regulated exon skipping in pregnant UA. The higher rate skipped exon events at the transcripts of *Traf3ip1* and *Abhd14b* and a lower rate of skipped exon events at the transcripts of *Asic5* and *Kif1a* are presented. The tracks represent pregnant (blue) and non-pregnant (red) samples. The number on curved lines indicates continuous (top number) and differentially-spliced (bottom number) exon-exon junction read counts. The x-axis depicts genomic coordinates. Inc level represents the exon inclusion level in pregnant and non-pregnant UA.

Among the observed 22,868 ASEs, 1882 events pertaining to 1686 genes showed differential alternative splicing (DASE) in pregnant compared to non-pregnant UA (at the threshold of FDR > 0.1, *p* > 0.05). Among the 1882 DASE events, 1154 SE (with 671 increased events in 597 genes and 883 decreased events in 781 genes), 239 MXE (with 105 increased events in 102 genes and 134 decreased events in 120 genes), 36 RI, 34 A3SS, and 17 A5SS were observed. Interestingly, 60 genes in pregnant UA showed both SE and MXE (with increased events in 25 genes and deceased events in 35 genes) (Table 3, Figure 7A,B, Supplementary Table S10). The top two representative rMATS sashimi plot for up (*Traf3ip1* and *Abhd14b)* and down (*Asic5* and *Kif1a)* exon skipping events in pregnant and non-pregnant UA are presented in Figure 8.

Furthermore, GO analysis to explore the biological process and cellular components impacted by these DASEs revealed that the spliced genes mainly participate in vascular microtubule dynamics. The top three biological processes were: regulation of microtubule-based process (12 genes), regulation of microtubule cytoskeleton organization (9 genes) and smooth muscle contraction (6 genes) (FDR = 0.05, *p* > 0.001). With regard to the cellular component, processes related to microtubule organizing center part (26 genes), microtubule organizing center (74 genes) and axon initial segment (3 genes) were impacted. In addition, the KEGG pathway analysis revealed that 19 genes in the phosphatidylinositol signaling system, 14 genes in the inositol phosphate metabolism, 18 genes in the autophagy were differentially spliced in pregnant UA compared with non-pregnant UA (FDR = 0.05, *p* > 0.05) (Supplementary Table S11).

### 2.7. Overlap of DEGs and DASEs

The DEGs and DASEs were together observed in 190 genes in the pregnant UA compared to non-pregnant UA. For 61 genes, their gene expression increased with an associated decrease in splicing in pregnant UA. On the other hand, for 40 genes, their expression decreased with an associated increase in splicing in pregnant UA (Figure 7C, Supplementary Table S12).

The EnrichR-GO analysis for the subset of genes that exhibited combined gene expression increase/splice level decrease (61 genes) showed higher enrichment for biological process: leukocyte and B cell biology (*Cd300a*, *Lgals9*, *Fcgr2b*, *Mnda* and *Fgr*) and cellular component: integral component of plasma membrane and endoplasmic reticulum lumen (*Adgre1*, *Abca1*, *Cd163*, *Cd4*, *Slc43a2*, *Ptpro*, *Fcgr2b*, *Tspan11*, *Csf2ra*, *Epha3*, *Ly6e*, *Eng*, *C4a*, *Vcan*, *Col16a1*, *Tnc*, *Fn1* and *Cp*). The corresponding KEGG pathway analysis identified the genes within this subset were members of osteoclast differentiation (*Ctsk*, *Fcgr2b* and *Lilrb4*), cholesterol metabolism (*Abca1* and *Soat1*) and PI3K-Akt signaling pathway (*Angpt4*, *Ccne2*, *Tnc* and *Fn1*) (Supplementary Table S13).

Similar analysis on the second subset of 40 genes that exhibited combined gene expression decrease/splice level increase showed enrichment for biological process: lipid modification, phosphorylation (*Dgkd* and *Dgkb*), wound healing (*Epb41l4b* and *Syt7*) and cellular process: early phagosome (*SYT7*), axonal growth cone (*L1CAM*), smooth endoplasmic reticulum (*RYR3*). The following KEGG pathway analysis showed that the genes within this subset are also involved in glycerolipid and glycerophospholipid metabolism (*Dgkd*, *Dgkb* and *Mboat2*), IL-17 signaling pathway (*Fosb* and *Il17re*) (Supplementary Table S13).

## 3. Discussion

To our knowledge, this is the first study exploring the whole-genome transcriptome differences between pregnant and non-pregnant uterine vasculature. This study identified differential transcriptional regulation of 2245 genes in the pregnant UA with activation of pathways related to the immune system and suppression of pathways involved in calcium signaling, AMPK signaling, and vascular smooth muscle contraction. We also revealed a unique gene cluster-specific transcription factor binding motifs that interact with DEGs. Differential exon skipping appears to be the most predominant alternative splicing event in UAs, with 190 genes exhibiting both differential gene expression and differential alternative splicing in the pregnant UA. Pathways related to cholesterol metabolism, PI3K-Akt signaling pathway, glycerophospholipid metabolism, and IL-17 signaling pathway exhibited both differential gene expression and differential alternative splicing. Furthermore, 13 novel upregulated genes (10–13 fold) including *Esm1*, *Slfn4*, *Usp18* and *Grem1* and 4 downregulated genes (4–7 fold), including *S100g*, *Pcp4*, *Col6a4,* and *Trpc3* were identified in the pregnant UA. While many of these genes were associated with gestational pathologies [34,35,36,37,38,39], the differential expression of these genes in healthy pregnancy relative to non-pregnancy suggests that these genes may have a unique role in gestational uterine vascular adaptations.

In mammals, the maternal vasculature undergoes exquisite remodeling during pregnancy, which allows the UA to withstand a 20–40 fold increase in blood flow compared to the non-pregnant state [3,40]. The clinical relevance of uterine vascular remodeling and reactivity is frequently emphasized by maladaptations contributing to gestational pathologies such as intrauterine growth restriction (IUGR) and preeclampsia [40,41,42,43,44]. It is widely accepted that the circumferential enlargement, along with axial elongation of the pregnant UA is mediated by substantial changes in several local genes [3]. Studies from our lab and others have shown explicit evidence for vascular specific transcriptional activation of unique genes (e.g., MMPs and TIMPs and estrogen receptor- ESR2) in gestational uterine vascular remodeling [45,46,47,48]. In the current study, the overall global changes in the uterine vasculature transcriptome activate a majority of pathways related to the local and peripheral immune response, which may help to tolerate the semi-allogeneic fetus [49]. Furthermore, the elevated immune responses in uterine vasculature may arise due to the extracellular matrix turnover by the recruitment of degradative cell types such as macrophages and natural killer cells, which are closely associated with elevated nitric oxide (NO) production [50,51,52,53]. Concurrently, we also noted the suppression of several pathways, including AMPK signaling and vascular smooth muscle contraction. Activation of AMPK pathway is known to restrict the vascular smooth muscle cell growth and their migration [54], thus the attenuation AMPK pathway in pregnant UA appears relavent since the cellular proliferation and migration, and matrix rearrangements are the essential steps in uterine vasculature vasculogenesis and angiogenesis [55]. Similarly, the increased synthesis and activity of NO, cGMP pathway and prostacyclin and other vasoactive peptides may attenuate the vascular smooth muscle contraction pathway in healthy pregnancy [56].

The perspective GO analysis for cellular function showed the activation of the DNA packaging complex, nucleosome and protein DNA complex and attenuation of glycoprotein complex, nuclear transcription factor complex, and acetyltransferase complex. Not surprisingly, these factors are known to regulate heterochromatin and euchromatin formation, which determines DNA accessibility to transcription factors, histone, and DNA modification like acetylation and methylation [57]. For example, estrogen specifically upregulates TET1 through promoter transactivation, which unleashes the KCNMB1 promoter CpG methylation at SP1 binding site, leading to enhanced BKCa channel β1 subunit expression which favors the uterine vascular adaptation during pregnancy [17]. We also recently showed a pregnancy-specific phenomenon where estrogen interacts with the ERβ promoter resulting in the enhanced expression AT2R—an important mediator of UA vasodilation [48].

Analysis in the context of molecular function showed the activation of peptidase activity, mainly serine-type peptidase, along with suppression of transferase activity, primarily the methyl-transferase activity. Endopeptidases are the enzymes that can cleave peptide bonds in proteins, which possess unique functions depending on their type and targets [58,59]. For example, serine-type endopeptidases are involved in the proteolytic cleavage of pro-atrial natriuretic peptide hormone [60], which promotes trophoblast invasion and spiral artery remodeling [61]. Similarly, chymotrypsin-like serine endopeptidases are major serine proteases in human arteries responsible for angiotensin-converting enzyme (ACE)-independent production of angiotensin II, which play a significant role in vascular remodeling and contraction [62]. MMPs are another group of endopeptidases that degrade various proteins in the extracellular matrix (ECM), including collagen and elastin [45], which determines vascular remodeling, angiogenesis, and the systemic changes in blood pressure in healthy pregnancy [45,63]. On the other hand, the attenuated UA methyltransferase activity, which is involved in DNA/RNA and histone methylation, is shown to contribute to the decrease in the KCNMB1 promoter CpG methylation in pregnant ovine UA [64].

The additional K means clustering analysis on the DEGs elucidated the hidden details, like the cluster of genes co-regulated together and that share the same cellular and molecular process and common regulatory motifs within each cluster. Our analysis indicates that the ETS, REL, and C2H2 ZF families of transcription factors may play a role in the upregulation of 851 genes since they share the common regulatory motifs and co-regulated as a cluster. Similarly, the transcription factors C2H2 ZF, AP-2 and CxxC may have a role in the downregulation of 788 genes, which also share common regulatory motifs in their promoters. Chemokines, cytokines, growth factors, and vasoactive peptides, which are enriched during pregnancy, are known to modulate the expression and activity of these transcription factors [65], which may then mediate the endothelial function and angiogenesis. For example, ETS-1 enhances the expression of MMPs, β3 integrin, vascular endothelial growth factor receptors and angiopoietin-2, to mediate endothelial migration and angiogenesis [66,67,68,69].

Precursor mRNA splicing is one of the essential steps in gene regulation. Based on demand and cellular need, the alternative splicing mechanisms are triggered in order to produce functionally diverse proteins from a single gene (isoforms), which may have an exclusive role in binding between proteins, ligands, nucleic acids or membranes, localization and enzymatic properties [70]. This process is well controlled by a dynamic and flexible macromolecular machine, including the splicing enhancers, silencers, serine-arginine rich proteins, and spliceosomes, which works in a synergistic and antistatic manner [70,71,72]. This study provides evidence for the pregnancy-specific differential splicing of 1686 genes in UA. The GO analysis suggests that these splicing events are implicated in processes related to vascular microtubule dynamics, which are important in the maintenance of vascular morphology and reactivity [73,74]. For example, microtubule is shown to regulate the subcellular localization of eNOS and its activity state (phosphorylation) [75,76]. Furthermore, microtubule depolymerization enhances endothelium-independent vascular smooth muscle cell contraction [75], while stabilized microtubules are known suppressors of vascular calcification [77].

In addition, our study discovered alternative splicing of VEGF-A and B, VEGFR1, VEGFR2, NRP-2, FGFRs, Vasohibin-1 and 2, HIF-1α, Angiopoietin-2, and Angiopoietin-4. Although these protein isoforms are identified in pathological conditions like cancer, Alzheimer’s disease and atherosclerosis, the mechanisms and significance of ASE-mediated production of isoforms in the pregnant UA are yet to be investigated. It is important to note that the above-mentioned protein isoforms are known to interact with the phosphatidylinositol signaling system, which has a unique physiological role in vascular endothelial and smooth muscle contraction [78,79,80,81,82,83]. Besides, this study revealed that differential splicing occurred in a small subset of DEGs (40 upregulated and 61 downregulated genes), suggesting that splicing may contribute to the differential expression of these genes. The decreased splicing is implicated in the increased expression of genes involved in leukocyte and B cell biology and integral component of the plasma membrane and endoplasmic reticulum functions. This is not surprising since leukocytes can bind and cross the endothelium and activate cellular signals [84,85,86,87]. Similarly, the increased splicing is implicated to contribute to the decreased expression of key genes involved in lipid modification and phosphorylation. For example, the lipid kinases family, diacylglycerol kinases convert diacylglycerol to phosphatidic acid to serve as a messenger to induce vascular cellular responses [88,89,90]. Future studies should examine if shifts of splice level relate to functional change. Similarly, whether diversification of splicing or switching to another splicing variant is more critical for pregnancy vascular adaptations needs to be clarified. Identified alternative splicing as a mechanism for regulating gene expression should be further investigated to explore their potential role in gestational adaptations and the possibility of their dysregulation in pathobiology.

## 4. Materials and Methods

### 4.1. RNA Sample Preparation from UA

All procedures of animal care and use were in accordance with National Institutes of Health guidelines (NIH Publication No. 85–23, revised 1996) with approval by the Institutional Animal Care and Use Committee at the University of Wisconsin at Madison. Twelve-week-old timed-pregnant (positive plug = gestation day 1) and non- pregnant Sprague-Dawley rats were purchased from Envigo laboratories and were maintained on 12 L/12 D cycles in a temperature-controlled room (23 °C) and provided with food (Teklad global rodent diet # 2020X) and water ad libitum. Total RNAs were extracted from the UA of concomitantly raised, non-pregnant (diestrus), and pregnant rats (gestational day 20, *n* = 3/group) using TRIzol (Thermo Fisher Scientific, Inc.) and subsequently purified using RNeasy kit (Qiagen) [41] according to the manufacturers’ instructions.

### 4.2. Library Preparation and Sequencing

The quality of all RNA samples was determined using a bio-analyzer (Agilent Technologies, Inc.) before the preparation of the sequencing library. An amount of ~1–2 μg of total RNA of each sample was used for RNA-seq library preparation [91]. Briefly, mRNA was enriched from total RNA with NEBNext Poly(A) mRNA Magnetic Isolation Module (NEB, #E7490S), and rRNA was removed with a RiboZero Magnetic Gold Kit (Epicenter, MRZG126). Following purification, the mRNA was fragmented into small pieces using divalent cations under elevated temperature. The cleaved RNA fragments were copied into first-strand cDNA using SuperScript II reverse transcriptase (Invitrogen) and random primers. This was followed by second-strand cDNA synthesis using DNA Polymerase I and RNase H. These cDNA fragments then went through an end repair process, the addition of a single ‘A’ base, and then ligation of the indexing adapters. The products were then purified and enriched with PCR to create the final cDNA library. The completed libraries were screened through Agilent 2100 Bioanalyzer (Agilent Technologies, Inc.) for concentration, fragment size distribution between 400~600 bp, and adapter dimer contamination and quantity was determined by absolute quantification qPCR method. The barcoded libraries were mixed in equal amounts and used for sequencing. The DNA fragments in well-mixed libraries were denatured with 0.1 M NaOH to generate single-stranded DNA molecules, loaded onto channels of the flow cell at 8 pM concentration, and amplified in situ using TruSeq SR Cluster Kit v3-cBot-HS (#GD-401-3001, Illumina). Sequencing was carried out using the Illumina Xten/NovaSeq according to the manufacturer’s instructions. Sequencing was carried out by running 150 cycles.

### 4.3. Data processing and Bioinformatics Analysis

The complete workflow pipeline for sequencing, data processing, differential expression, alternative splicing, and bioinformatics analysis is presented in Figure 1. Image analysis and base calling were performed using Solexa pipeline v1.8 (Off-Line Base Caller software, v1.8). The raw paired-end reads quality was verified using the FastQC software. The trimmed reads (trimmed 5′, 3′-adaptor bases using cutadapt) were aligned to the reference genome using Hisat2 software. The transcript abundances for each sample was estimated with StringTie, and the FPKM value for gene and transcript level were calculated with R package Ballgown. The differentially expressed genes and transcripts were filtered using R package Ballgown. The novel genes and transcripts were predicted from assembled results by comparing to the reference annotation using StringTie and Ballgown, then use CPAT to assess the coding potential of those sequences [92]. Then, rMATS was used to detecting alternative splicing events [93]. Principle Component Analysis (PCA) and correlation analysis were based on the gene expression level in R, Python, or shell environment for statistical computing and graphics. K means clustering, gene ontology, pathway analysis, were performed with the differentially expressed genes using iDEP (integrated Differential Expression and Pathway analysis) using R/Bioconductor packages along with comprehensive annotation and pathway databases for humans and animals [94].

### 4.4. qPCR Validation of DEGs

Quantitative polymerase chain reaction (qPCR) was performed on the top 4 up- and down-regulated genes from the RNA sequencing data set. One microgram of total RNA from pregnant and non-pregnant UA (*n* = 3/ group) was reverse transcribed using an iScript cDNA synthesis kit (Bio-Rad Laboratories, Hercules, California), and real-time reverse transcription PCR was performed using iTaq Universal SYBR Green Supermix (Bio-Rad) with gene-specific primers for *Esm1*, *Slfn4*, *Usp18, Grem1*, *S100g*, *Pcp4*, *Col6a4* and *Trpc3.* Primers were designed based on the Ensembl Rat genome version Rnor_6.0 using Primer3 and purchased from Integrated DNA Technologies (Coralville, Iowa; Table 1). Results were calculated using the 2^−ΔΔ*C*T^ method and expressed as fold changes in the treatment group versus control. All reactions were performed in duplicate, and *Gapdh* was used as an internal control.

## 5. Conclusions

This is the first study that used high throughput next-generation RNA sequencing to identify differential gene expression and splicing profile in pregnant UA compared with non-pregnant UA. Our study provides a valuable resource for the understanding of the molecular mechanisms underlying normal gestational vascular adaptations, which could also be useful for predicting the vascular signaling pathways involved in IUGR and preeclampsia. Our data suggest that the observed alternative splicing events may produce protein isoforms that may have a unique role in pregnant UA adaptations. How the prevailing conditions like pregnancy dictate which isoform is to be expressed remains an area requiring further exploration.

## Figures and Tables

**Table 1:** Summary of UA mRNA-Sequencing.

Samples	Raw Pairs	Trimmed	Mapped	Unmapped
P-1	81682276	81349316	91.10%	8.90%
P-2	62957576	62868673	90.68%	9.32%
P-3	73435461	73393727	90.98%	9.02%
NP-1	74182993	74137154	91.43%	8.57%
NP-2	54768093	54662400	90.24%	9.76%
NP-3	61775409	61716471	91.96%	8.04%

mRNA of UA from pregnant and on pregnant rats (*n* = 3/group) were sequenced on the Illumina X-ten/NovaSeq platform, yielding approximately 50–80 million 2 × 125-bp paired-end reads per sample, which were then mapped to the rat reference genome (RNO6 version). NP-non-pregnant; P-pregnant.

**Table 2:** Top five pathways from each cluster, p-values, and the total number of genes involved in each pathway.

Cluster	Pathways	adj.Pval	nGenes
A	Calcium signaling pathway	7.36 × 10^−7^	25
A	Protein digestion and absorption	7.36 × 10^−7^	17
A	Neuroactive ligand-receptor interaction	5.20 × 10^−6^	33
A	Adrenergic signaling in cardiomyocytes	1.78 × 10^−5^	19
A	Vascular smooth muscle contraction	1.95 × 10^−5^	18
B	Complement and coagulation cascades	1.22 × 10^−19^	15
B	Chemical carcinogenesis	7.72 × 10^−12^	10
B	Steroid hormone biosynthesis	7.17 × 10^−11^	9
B	Cholesterol metabolism	1.19 × 10^−8^	7
B	Linoleic acid metabolism	5.69 × 10^−08^	6
C	Phagosome	5.49 × 10^−15^	35
C	Cell adhesion molecules (CAMs)	1.00 × 10^−12^	31
C	Antigen processing and presentation	2.46 × 10^−12^	22
C	Viral myocarditis	2.63 × 10^−11^	20
C	Tuberculosis	3.07 × 10^−11^	30
D	Systemic lupus erythematosus	2.42 × 10^−15^	18
D	Viral carcinogenesis	7.93 × 10^−10^	18
D	Epstein-Barr virus infection	1.30 × 10^−8^	17
D	African trypanosomiasis	2.90 × 10^−7^	8
D	Complement and coagulation cascades	5.26 × 10^−7^	10

Four Unique clusters were identified by K-means clustering, and the top 5 pathways from each cluster are presented in the table.

**Table 3:** Summary of the numbers of five types of ASE and significant upregulated and downregulated events.

ASE Type	No. of Events (%)	Number of Genes Involved	Significant Number of Events (Up/ Down)	Significant Number of Genes (Up/Down)
A5SS	160 (0.69)	152	17 (6/11)	17 (6/11)
A3SS	273 (1.19)	247	35 (20/15)	35 (20/15)
MEX	2533 (11.07)	1647	239 (105/134)	222 (102/120)
SE	19707 (86.17)	7747	1554 (671/883)	1378 (597/781)
RI	195 (0.85)	178	37 (6/31)	34 (6/28)
Total	22868 (100)	9971	1882	1686

## Supplementary Materials

Supplementary materials can be found at https://www.mdpi.com/1422-0067/21/6/2079/s1. Supplementary Figure S1. Q30 quality scores for all samples. Supplementary Figure S2. Principal component (PCA) and Pearson R2 gene expression correlation analysis. Supplementary Table S1. Quality score for the RNA seq. Supplementary Table S2. Genes identified with positive transcriptional signal (FPKM reads) in pregnant and non-pregnant UA. Supplementary Table S3. Transcripts identified with positive transcriptional signal (FPKM reads) in pregnant and non-pregnant UA. Supplementary Table S4. Differentially expressed Genes between pregnant and non-pregnant UA. Supplementary Table S5. Altered GO: Cellular component and Molecular function between pregnant and non-pregnant UA. Supplementary Table S6.Altered KEGG pathways between pregnant and non-pregnant UA. Supplementary Table S7. Differentially expressed gene clusters between pregnant and non-pregnant UA. Supplementary Table S8. TRUSTR transcription factor motif enrichment for differentially expressed gene clusters between pregnant and non-pregnant UA. Supplementary Table S9. Evidence for different types of alternative splicing events (ASE) in pregnant and non-pregnant UA. Supplementary Table S10. Differentially spliced genes between pregnant and non-pregnant UA. Supplementary Table S11. Gene Ontology and KEGG pathways for differentially spliced genes between pregnant and non-pregnant UA. Supplementary Table S12. Overlap of DEGs and DASEs between pregnant and non-pregnant UA. Supplementary Table S13. Gene ontology and KEGG pathways for splicing up/down and gene expression up/down between pregnant and non-pregnant UA. RNA Sequencing data availability: The RNA sequencing data from this study have been deposited in to Gene Expression Omnibus (GEO) Sequence Read Archive (SRA) with accession number GSE143327.

## Abbreviations

UA	Uterine Arteries
PGESA	Pre-Ranked Gene Set Enrichment Analysis
TF	Transcription Factor
GO	Gene Ontology
DEGs	Differentially-Expressed Genes
ASE	Alternative Splicing Events
DASE	Differential Alternative Splicing Events
