# Supplementary figures and images for: Whole-Genome Uterine Artery Transcriptome Profiling and Alternative Splicing Analysis in Rat Pregnancy
Source: Int J Mol Sci. 2020 Mar 18;21(6):2079. doi: 10.3390/ijms21062079 (PMC7139363; doi:10.3390/ijms21062079)

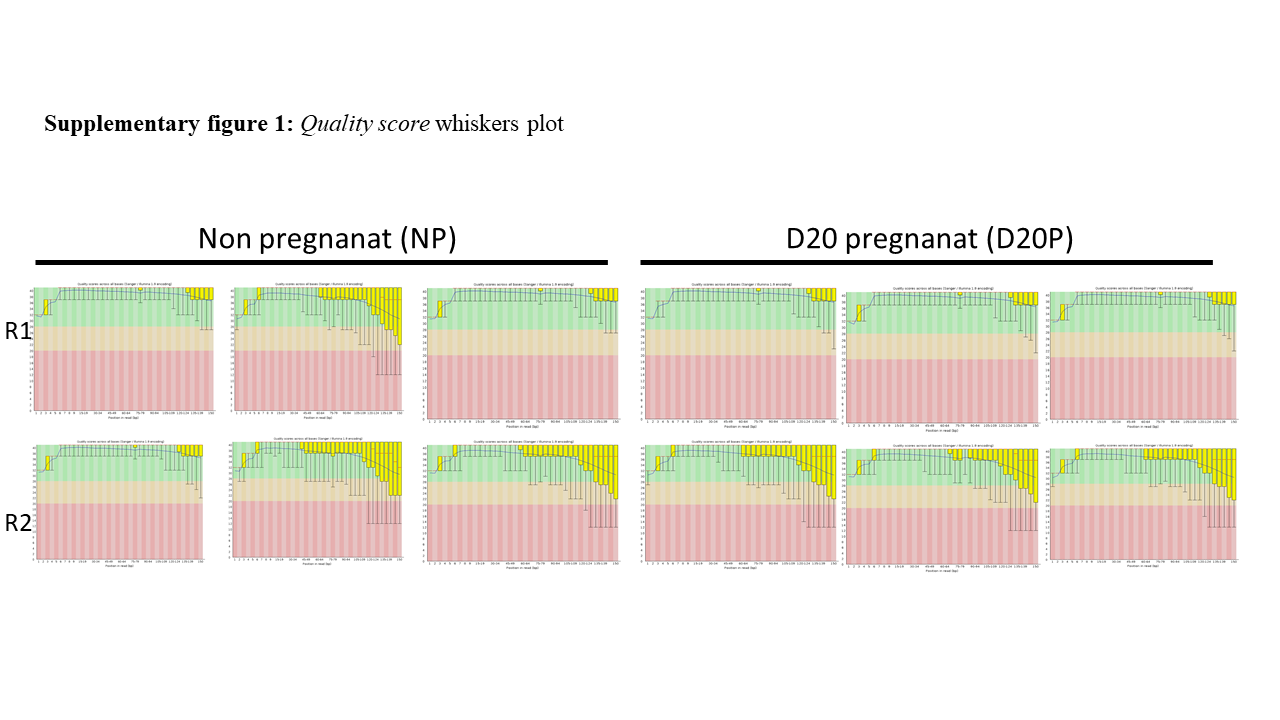

Supplement: Supplementary file 1 [file ijms-21-02079-s001.zip › Supplementary tables and figures/Supplementary figure 1.tif]

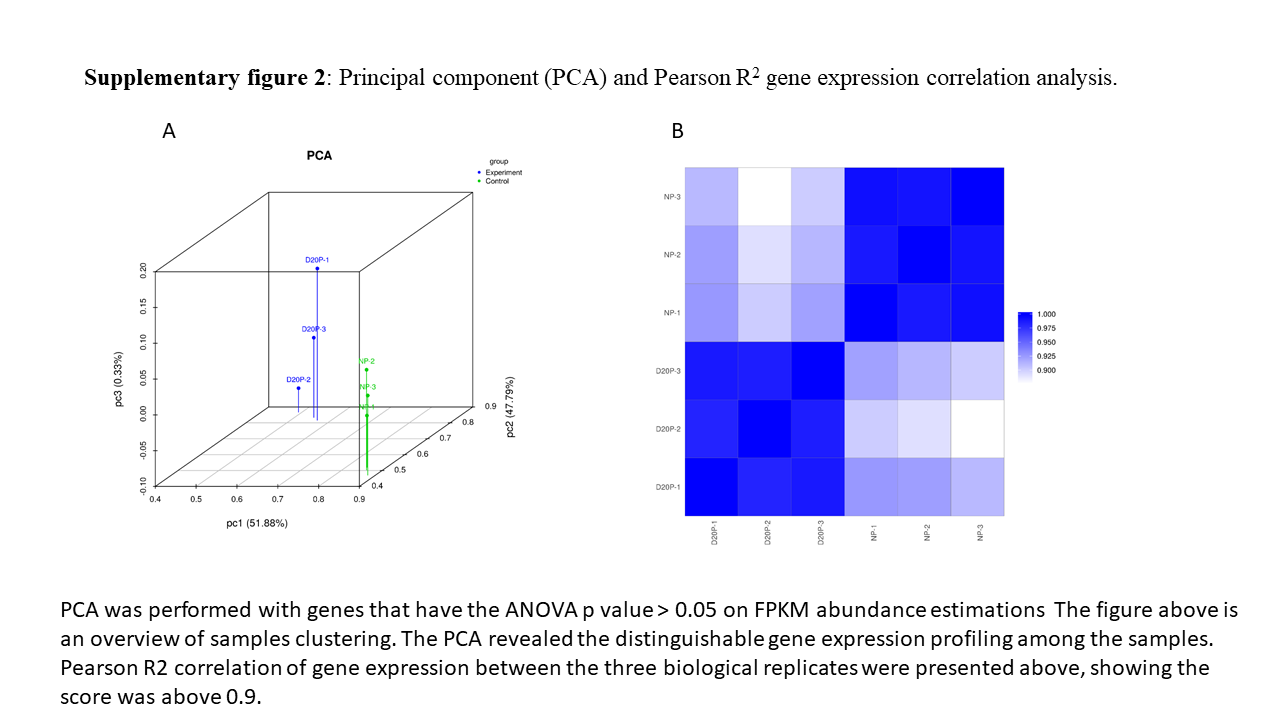

Supplement: Supplementary file 1 [file ijms-21-02079-s001.zip › Supplementary tables and figures/Supplementary figure 2.tif]
